# Editorial: Cellular and molecular targets in epileptogenesis focusing on disease prevention

**DOI:** 10.3389/fncel.2023.1251038

**Published:** 2023-07-12

**Authors:** Diana Cunha-Reis, Sandra Henriques Vaz, Paulo Correia-de-Sá

**Affiliations:** ^1^Biosystems and Integrative Sciences Institute (BioISI), Faculdade de Ciências, Universidade de Lisboa, Lisboa, Portugal; ^2^Departamento de Biologia Vegetal, Faculdade de Ciências, Universidade de Lisboa, Lisboa, Portugal; ^3^Instituto de Medicina Molecular João Lobo Antunes, Faculdade de Medicina, Universidade de Lisboa, Lisboa, Portugal; ^4^Instituto de Farmacologia e Neurociências, Faculdade de Medicina, Universidade de Lisboa, Lisboa, Portugal; ^5^Laboratório de Farmacologia e Neurobiologia, Instituto de Ciências Biomédicas de Abel Salazar (ICBAS), Universidade do Porto (UP), Porto, Portugal; ^6^Center for Drug Discovery and Innovative Medicines (MedInUP), Porto, Portugal

**Keywords:** epileptogenesis, miRNA, DNA methylation, synaptic plasticity (LTP/LTD), blood-brain barrier, inflammation, seizures-diagnosis, mesial temporal lobe epilepsy (MTLE)

Epilepsy is a complex disease characterized by the development of recurrent, unprovoked seizures, often associated with comorbidities, such as cognitive deficits, depression, anxiety and psychiatric disturbances, that worsen the patient's condition and mortality (Devinsky et al., 2018; Vezzani et al., 2019). Temporal lobe epilepsy (TLE) is the most common type of partial epilepsy in adulthood. Its most prevalent mesial form is characterized by seizures originating mostly in the hippocampus, but also in the amygdala and parahippocampal gyrus. Mesial-temporal lobe epilepsy (MTLE) is often accompanied by hippocampal sclerosis (MTLE-HS), commonly associated with antiseizure drug (ASD) resistance (Sloviter, 2005; Thom, 2014; Gambardella et al., 2016). The mechanisms underlying MTLE epileptogenesis are largely unknown, yet associated hereditary and environmental triggering factors, such as trauma, complex febrile seizures, *status epilepticus* (*SE*), inflammatory insults, or ischemia, are frequently detected. Disease onset and progression vary considerably with the putative triggering event and age. Several cellular mechanisms contribute to early epileptogenesis, such as altered synaptic plasticity and neuronal excitability, neuronal death, astrogliosis, and astrocyte dysfunction, neuroinflammation, blood-brain barrier leakage, secondary non-convulsive *SE* and aberrant neurogenesis, yet their pathophysiological relevance is far from being understood. At least one-third of epileptic cases have no available medical treatment. This prompted the focus of research toward the understanding epilepsy etiopathogenesis and to the development of novel therapeutic strategies aiming at epileptogenesis prevention (Pitkänen et al., 2015; Cunha-Reis et al., 2021). This Research Topic highlights the discovery of new circulating biomarkers for early diagnosis and follow-up of epileptic cases and addresses new therapeutic strategies to mitigate early cellular events and the progression of epilepsy to avoid ASD resistance.

Pioneering studies showed that blood-brain barrier (BBB) breakdown generates a vicious cycle favoring epileptic seizures, while seizure-induced BBB disruption is critical to epilepsy onset and progression. Using the pilocarpine-induced *SE* model in the rat, the article by Mendes et al. shows that altered BBB permeability to small molecules occurs within the first 4 h of *SE*, preceding macromolecule leakage in the following 5 h. While BBB leakage of macromolecules disappears within 24h, increased BBB permeability to small molecules persists, adding to brain damage over time. Therefore, the authors propose that there is a critical 24h temporal window of BBB dysfunction after the onset of the *SE*, during the acute phase, where therapeutic approaches should concentrate to prevent BBB damage and epileptogenesis.

Early and delayed BBB disruption leads to leakage of brain contents, which may provide biomarkers for the early diagnosis and progression monitoring of epileptogenesis. The article by Martins-Ferreira et al., reviews the putative role of methylated circulating cell-free DNA generated by neuronal apoptosis as a biomarker of ongoing epileptogenesis. Such an approach, relying on the cell-type specificity of DNA methylation (DNAm) to determine its tissue origin, is not currently used to approach epilepsy. Though it might be interesting, further studies are required to overcome the main limitations, such as the fact that early epileptogenesis depends on the triggering event and might not concur with neuronal apoptosis and cell death (Pitkänen et al., 2015). In line with this, Berger et al. review the role of DNAm changes as a trigger for early epileptogenesis alterations in gene expression (GE), extrapolating mainly on discoveries using the intracortical kainic acid rat model. The authors emphasize the role of glial cell DNAm in controlling early GE alterations involved in the regulation of neuronal death, reactive astrogliosis and brain inflammation and discuss how these processes could be targeted for the early prevention of epileptogenesis through directed epigenetic modifications.

Baloun et al. performed a cross-sectional study using hippocampal tissue from of MTLE-HS patients and from the Li^2+^-pilocarpine TLE animal model to uncover distinct miRNA profiles depending on the age of epilepsy onset in both patients and animal models. This analysis revealed overlapping miR-142-5p and miR-129-2-3p changes between MTLE-HS patients and rats with adult TLE onset. These miRNAs regulate immunomodulatory agents with convulsive and neuronal growth suppression properties that may be used in diagnosis. The study by Leal et al. extends this concept showing that low serum levels of circulating miRNA-22 correlate with overexpression of the proconvulsant ATP-sensitive ionotropic P2X7 receptor in the hippocampus and neocortex of MTLE-HS patients. These changes, which are more notorious in patients' refractory to three or more ASDs, seem to occur soon after the epileptogenic trigger and are not dependent on the age of onset and gender, making them useful as predictors of drug refractoriness. Interestingly, two P2X7 isotypes were identified in hippocampal and neocortical nerve terminals, being the higher MW (85KDa) isoform the most abundant in brain regions of MTLE-HS patients compared to the naturally occurring 67 kDa receptor. This may denote post-translational protein modifications, which epileptogenesis implications are worth investigating in the future.

Carvalho-Rosa et al., used *in vitro* models of epileptiform activity (EA) to evaluate the time course of long-term potential (LTP) changes occurring within 30 min to 1 h 30 m following EA while characterizing the early modifications in synaptic structure leading to altered synaptic transmission patterns and neuronal excitability that may contribute to early epileptogenesis. The described synaptic molecular alterations in AMPA GluA1/GluA2 levels and AMPA GluA1 phosphorylation likely underlie the observed impaired post-seizure LTP and, notably, were associated modifications in synaptic lipid raft structure. Since lipid raft integrity is required for several molecular mechanisms involved in synaptic metaplasticity these may constitute promising targets for prevention of epileptogenesis.

Zhang C. et al. show that altered expression of Par3, αPKC-λ, and Lgl1 proteins and enzymes participating in neuronal polarity definition and axonal growth during development are correlated with mossy fiber sprouting and neuronal cell loss in the CA3 region of the hippocampus from 3 days after kainic acid (KA)-induced SE in rats. While these findings suggest the involvement of Par3, aPKC-λ, and Lgl1 in early epileptogenesis, their clinical translation requires further investigation. The group of Zhang S. et al. reviewed preclinical studies and clinical evidence concerning the role of two glia-to-neuron signaling pathways, the high mobility group box-1 (HMGB1)/toll-like receptor 4 (TLR4) and interleukin-1beta (IL-1β)/interleukin-1 receptor 1 (IL-1R1) pathways in the neuroinflammatory response contributing to brain injury in epilepsy. Although these signaling pathways were subject to investigation as therapeutic targets through the development of antibodies and inhibitors, the complete upstream and downstream links are still missing.

Finally, the articles by Schulze et al., Tse et al., and Zhang M. et al. go a step further by testing the impact of distinct therapeutic strategies in epileptic animal models. The first paper demonstrates the role of casein kinase 2 (CK2) activity in epileptogenesis in juvenile rats. Inhibition of CK2 activity before SE had a neuroprotective role on seizure onset, disease progression and chronic CA1 neuronal burst firing, by increasing K_Ca_2.2 levels and function, also involving the upregulation of HCN1 and HCN3 channels. Tse et al. demonstrated that surgical implantation of depth electrodes increased brain and plasma molecules involved in epileptogenesis and neuroinflammation while it reduced the threshold for *SE* induced by KA. Implantation chronology aside, this suggests that electrode implantation in TLE patients may contribute to neuroinflammation, neurodegeneration and BBB leakage at the risk of accelerating disease progression. Zhang M. et al. report the neuroprotective role of subclinical anesthetic doses of xenon gas against epileptogenesis in the pentylenetetrazole (PTZ) kindling model of TLE. These actions were evident early in the development of epileptogenesis and were probably due to the reduction of iron and oxidative and iron stress.

In summary, this Research Topic puts into evidence multiple targets for the early diagnosis as well as therapeutic interventions directed at epileptogenesis highlighting its benefits, caveats, and hurdles to clinical applications.

## Author contributions

DC-R: funding acquisition and writing—original draft, review, and editing. SV and PC-d-S: writing—review and editing. All authors contributed to the article and approved the submitted version.
